# Supplementary material for: Activating an anterior nucleus gigantocellularis subpopulation triggers emergence from pharmacologically-induced coma in rodents
Source: Nat Commun. 2019 Jul 1;10:2897. doi: 10.1038/s41467-019-10797-7 (PMC6603023; doi:10.1038/s41467-019-10797-7)
Supplement: Supplementary file 1 — Supplementary Information [file 41467_2019_10797_MOESM1_ESM.pdf]

Activating an anterior nucleus gigantocellularis subpopulation triggers emergence from pharmacologically-induced coma in rodents

Gao et al.

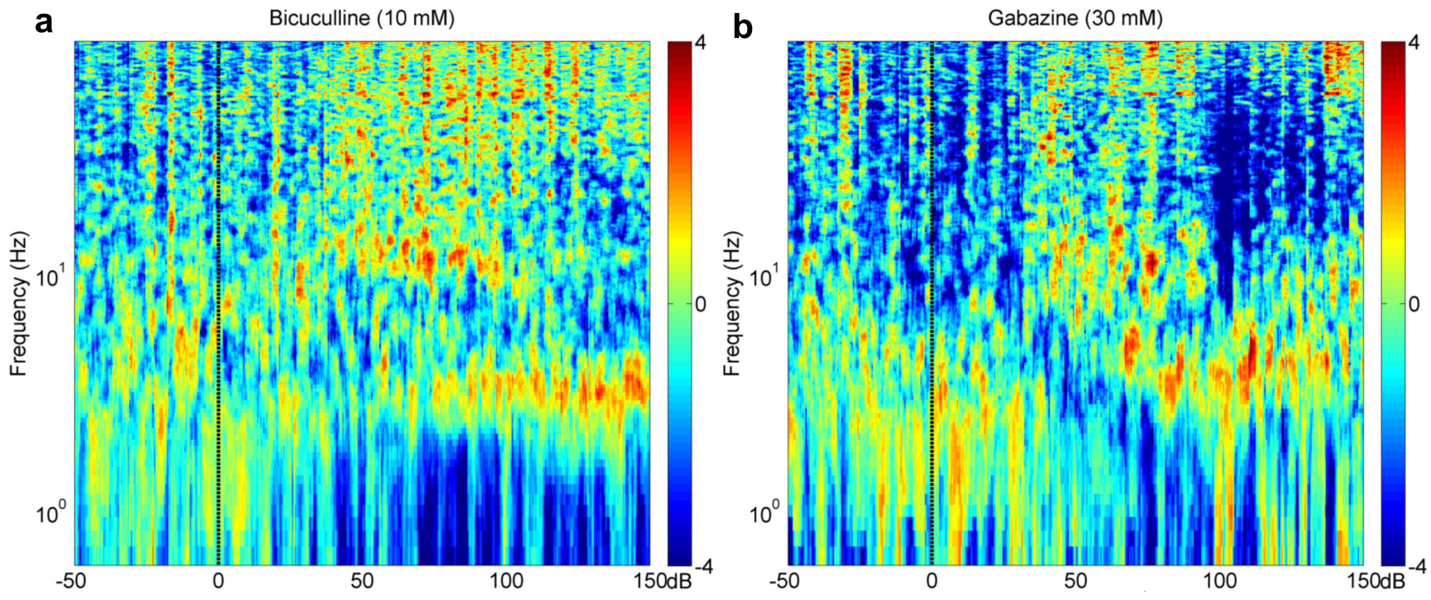

Supplementary Figure 1. Specific GABAa receptor antagonist, Gabazine produces similar cortical activation as bicuculline. Graph shows an average power spectrogram obtained from cingulate cortex of animals injected in aNGC with (**a**) bicuculline(n=5) and (**b**) Gabazine (n=4). Color bar represents power in decibels (dB). In both cases, there is a transient disappearance of slow oscillations and a predominance of high frequencies after drug application at time zero (dashed lines).

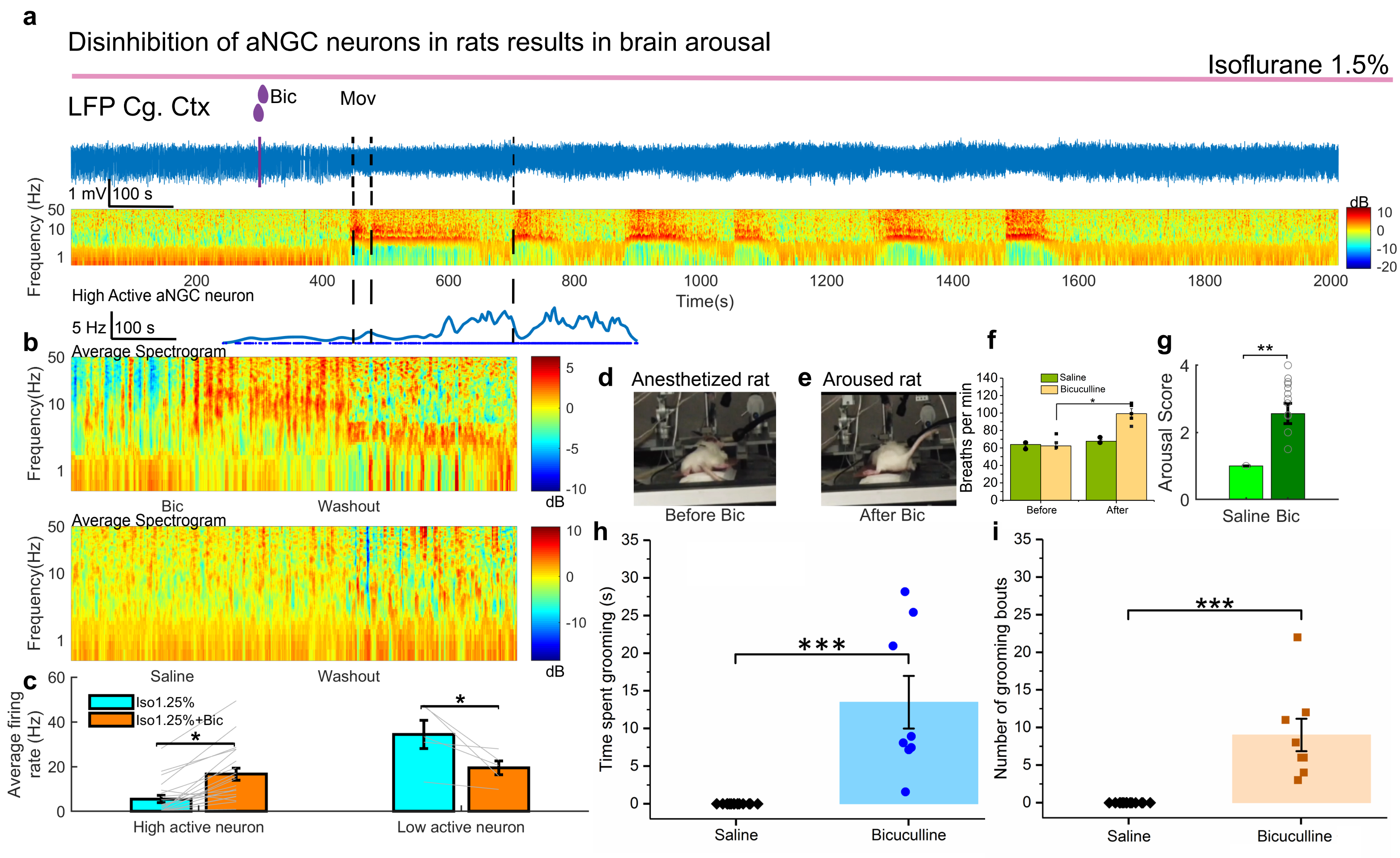

Supplementary Figure 2. Disinhibition of aNGC neurons in rats facilitates cortical, autonomic, and behavioral arousal. (**a**) Cortical LFPs (raw data) and normalized power spectrogram (deviation from mean) show increased power of high frequency oscillations seconds after microinjection of bicuculline (Bic;purple drops) in aNGC under a constant concentration of isoflurane 1.5% vol. for 1 h. Firing rate of a representative aNGC neuron (bottom panel) increased prior to changes in cortical activity and firing rate remained high during movement (dashed line). (**b**) Average spectrogram relative to bicuculline (top; n=8) or saline (bottom; n=3). Color bar represents power in decibels(db). (**c**) Average firing rate obtained from high active (HA;n=19) and low active neurons (LA;n=6) before and after microinjection of bicuculline and 25 minutes later (washout).
Data are represented as mean+/- s.e.m. Firing rate data was analyzed using two-way ANOVA*p=<0.05. The population means between cell types were significantly different F=(1,1), 11.22 ,p=0.001. (**d**) Animal displays loss of righting reflex during PIC (isoflurane 1.5% vol.).(**e&g**) After disinhibition of aNGC cells, animals increased arousal (see methods) and righted (Mann-Whitney U-test, **p=<0.01).
(**f**) Breaths per minute increased t-test *p=<0.05. (**h&i**). Animals with an arousal score >3 showed grooming behavior (paired t-test, ***p=<0.001).

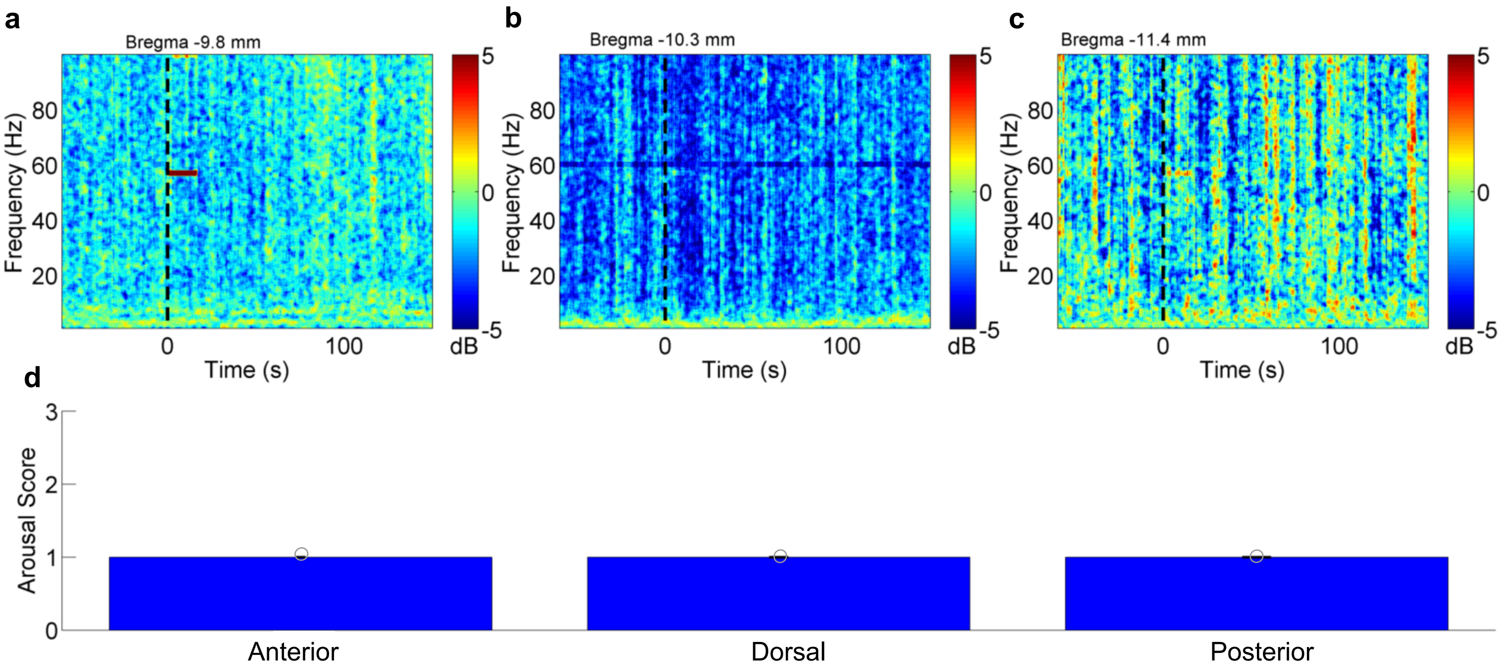

Supplementary Figure 3. Anatomical boundaries of the gigantocellular responsive area. **(a)** Average spectrogram of (n=3) injections in the anterior side of aNGC. This area is defined by the following coordinates: AP:≥ -9.8 mm DV: -7.0 to -8.2 mm ML: 0.7 mm from bregma. Color bar represents power in decibels (dB). Note the lack of changes in the spectrogram when GABAa antagonist, bicuculline(10 mM) is injected in this area (dashed line). (**b**) Average spectrogram of (n=3) injections located in the dorsal side. This area is defined by the following coordinates: AP:-10.3 mm DV: -6.2 to-7.5 mm and ML:0.7 mm from bregma. (**c**) Average spectrogram (n=3) post-GABAa antagonist injection in the posterior side of aNGC. This area is defined by the following coordinate: AP≤ -11.4 mm from bregma. (**d**) Arousal score of animals with injections located in anterior, dorsal and posterior sides of aNGC. Scores obtained from two researchers blind to the experiment.

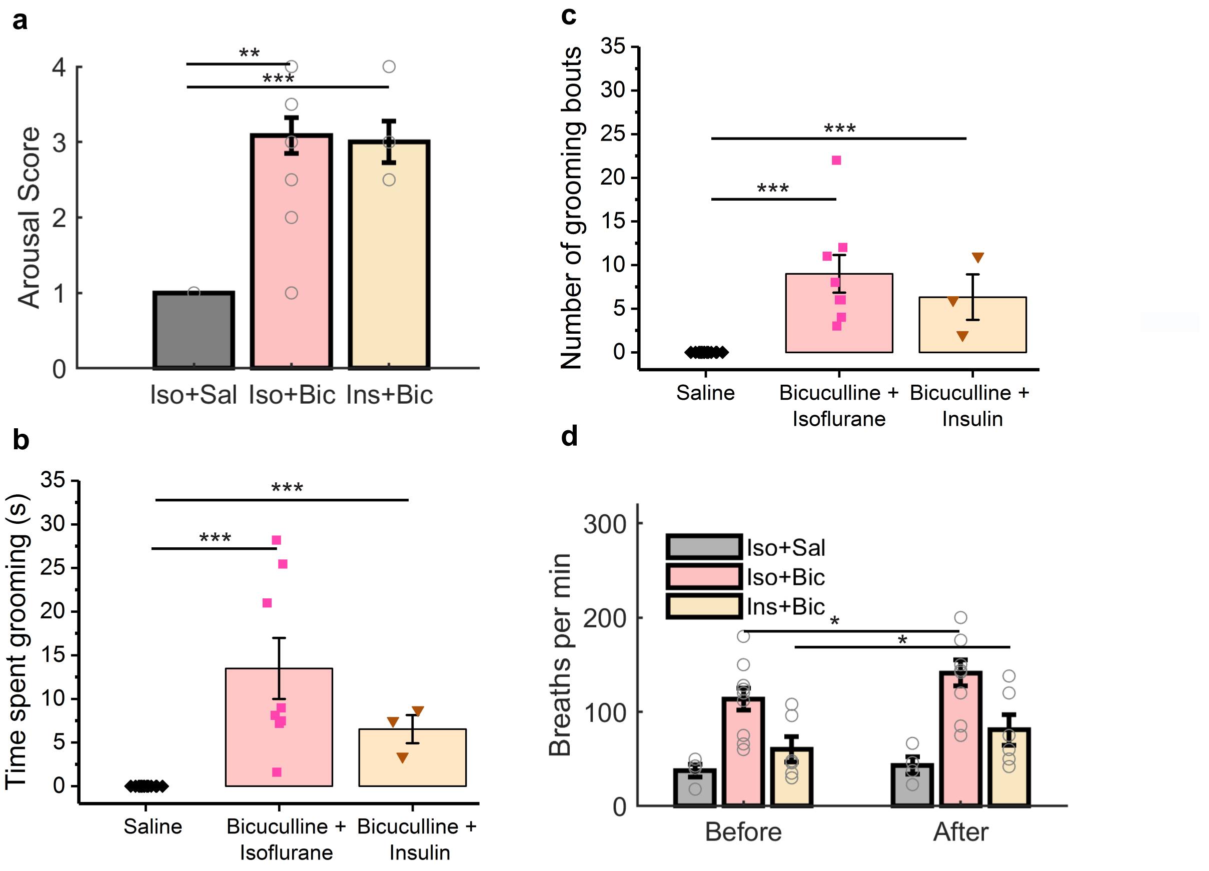

Supplementary Figure 4. Behavioral responses in anesthetized and hypoglycemic coma mice after aNGC stimulation (**a**) Quantification of organized movements using an arousal scale to score videos of animals injected with Bic or saline (Sal) (n=6) in anesthetized (n=17) and hypoglycemic mice (n=6). KST, saline vs. iso+Bic: **p=<0.01 & saline vs. Ins+Bic: ***p=<0.001. (**b&c**) Animals showed grooming behavior (paired t-test, ***p=<0.001). (**d**) Respiratory frequency increased compared to saline (n=4) and before and post-injection in isoflurane (n=10) and hypoglycemic coma (n=6;t-test: *p=<0.05).
