# Supplementary material for: Activating an anterior nucleus gigantocellularis subpopulation triggers emergence from pharmacologically-induced coma in rodents
Source: Nat Commun. 2019 Jul 1;10:2897. doi: 10.1038/s41467-019-10797-7 (PMC6603023; doi:10.1038/s41467-019-10797-7)
Supplement: Supplementary file 8 — Description of Additional Supplementary Files [file 41467_2019_10797_MOESM8_ESM.docx]

Description of Additional Supplementary Files

**Supplementary Movie 1** This video shows organized vigorous body movements and increased respiratory rate in rats and mice that were injected with GABAa antagonist in aNGC during constant exposure to anesthetic isoflurane (1.5% rats and 1.25 mice).

**Supplementary Movie 2** This video shows a hypoglycemic comatose mouse that was unresponsive to pinching. After a brief train of light pulses activating Vglut2+cells, the subject became responsive to painful stimuli. The subject displays brief movement of tail and limbs with short trains of light pulses. Prolonged laser pulses resulted in recovering an upright posture.

**Supplementary Movie 3** This video shows motor arousal and increased respiratory rate after photoinhibition of GABA ergic aNGC-cells using dual optic fiber. GA stands for gigantocellular area.

**Supplementary Movie 4** This video shows an unrestrained animal exposed to a constant concentration of isoflurane (1.25%) exhibiting grooming, elliptical strokes and scratching after acute injection of bicuculline via a cannula previously implanted in aNGC.

**Supplementary Movie 5** This video shows the response of an animal to a mechanical stimulation (pinch) before and after bicuculline microinjection in aNGC in a mouse experiencing hypoglycemic coma.

**Supplementary Movie 6** This video shows the response of an animal to mechanical stimulation (pinch) or chemical stimulation(odor) after a light pulse activating aNGC Vglut2+cells.
