# Supplementary material for: Activating an anterior nucleus gigantocellularis subpopulation triggers emergence from pharmacologically-induced coma in rodents
Source: Nat Commun. 2019 Jul 1;10:2897. doi: 10.1038/s41467-019-10797-7 (PMC6603023; doi:10.1038/s41467-019-10797-7)
Supplement: Supplementary file 10 — Reporting Summary [file 41467_2019_10797_MOESM10_ESM.pdf]

nature research

Corresponding author(s): Diany Paola Calderon

Reporting Summary

Nature Research wishes to improve the reproducibility of the work that we publish. This form provides structure for consistency and transparency in reporting. For further information on Nature Research policies, see Authors & Referees and the Editorial Policy Checklist.

Statistical parameters

When statistical analyses are reported, confirm that the following items are present in the relevant location (e.g. figure legend, table legend, main text, or Methods section).

n/a	Confirmed	
☐	☒	The exact sample size (*n*) for each experimental group/condition, given as a discrete number and unit of measurement
☐	☒	An indication of whether measurements were taken from distinct samples or whether the same sample was measured repeatedly
☐	☒	The statistical test(s) used AND whether they are one- or two-sided<br>*Only common tests should be described solely by name; describe more complex techniques in the Methods section.*
☐	☒	A description of all covariates tested
☐	☒	A description of any assumptions or corrections, such as tests of normality and adjustment for multiple comparisons
☐	☒	A full description of the statistics including central tendency (e.g. means) or other basic estimates (e.g. regression coefficient) AND variation (e.g. standard deviation) or associated estimates of uncertainty (e.g. confidence intervals)
☐	☒	For null hypothesis testing, the test statistic (e.g. *F*, *t*, *r*) with confidence intervals, effect sizes, degrees of freedom and *P* value noted<br>*Give P values as exact values whenever suitable.*
☒	☐	For Bayesian analysis, information on the choice of priors and Markov chain Monte Carlo settings
☒	☐	For hierarchical and complex designs, identification of the appropriate level for tests and full reporting of outcomes
☒	☐	Estimates of effect sizes (e.g. Cohen's *d*, Pearson's *r*), indicating how they were calculated
☐	☒	Clearly defined error bars<br>*State explicitly what error bars represent (e.g. SD, SE, CI)*

Our web collection on statistics for biologists may be useful.

Software and code

Policy information about availability of computer code

Data collection	Plexon, Spike2 (CED)
Data analysis	functions written in Matlab has been described in the paper

For manuscripts utilizing custom algorithms or software that are central to the research but not yet described in published literature, software must be made available to editors/reviewers upon request. We strongly encourage code deposition in a community repository (e.g. GitHub). See the Nature Research guidelines for submitting code & software for further information.

Data

Policy information about availability of data

All manuscripts must include a data availability statement. This statement should provide the following information, where applicable:

- Accession codes, unique identifiers, or web links for publicly available datasets
- A list of figures that have associated raw data
- A description of any restrictions on data availability

The data that support the findings of this study are available from the accession code provided in the manuscript or the corresponding author

Field-specific reporting

Please select the best fit for your research. If you are not sure, read the appropriate sections before making your selection.

☒ Life sciences ☐ Behavioural & social sciences ☐ Ecological, evolutionary & environmental sciences

For a reference copy of the document with all sections, see nature.com/authors/policies/ReportingSummary-flat.pdf

Life sciences study design

All studies must disclose on these points even when the disclosure is negative.

Sample size	To determine the sample sizes of the experimental groups we performed pilot experiments with 3 mice for the pharmacologic and optogenetic experiments. We considered the strength of the effect and the variance across the groups to determine the sample size (number of units). For experiments in which we assessed arousal, grooming or breathing frequency, we estimated samples sizes using data previously published16,47,61. In iDISCO experiments we estimated sample size considering previous data published by one of the authors33. We estimated ideal samples by conducting power analysis. All experiments met or exceeded ideal sample size.
Data exclusions	*Describe any data exclusions. If no data were excluded from the analyses, state so OR if data were excluded, describe the exclusions and the rationale behind them, indicating whether exclusion criteria were pre-established.*
Replication	In the iDISCO-cFIR experiments, the data obtained was replicated 3 times. Each of these sets of experiments had at least 3 animals injected with bicuculline vs. 3 using saline(vehicle). Other experiments were replicated in several animals
Randomization	In optogenetic experiments, Vglut2-Cre mice or Gad-Cre mice were randomly assigned to control (injected with AAV-EF1a-DIO-mcherry or AAV-CAG-FLEX-GFP) and experimental groups (injected with AAV-EF1a-DIO-hChR2(H134R)-mCherry or AAV-CAG-FLEX-ArchT-GFP). In pharmacolologic experiments mice and rats were randomly assigned to micro injection of bicuculline or saline.<br>aNGC imaging: We took images from random positions within aNGC using an Axiocam 506 monochromatic camera connected to an Axio Zoom V16 Stereo Zoom microscope from Zeiss.
Blinding	In the iDISCO-cFIR experiments, an investigator not involved in the experimental procedure blindly ran the algorithm for quantification. In arousal assessment, Arousal responses to bicuculline in rats and mice were obtained during dark cycle and characterized by a pre-established scale to assess arousal responses. Data is the result of averaging the score of two trained researchers that were unaware to the experimental conditions and exposed to videos showing the motor behavior. Grooming behavior was quantified by a trained researcher who was unaware to the experimental conditions and exposed to videos showing grooming. In pharmacologic and optogenetic experiments, an investigator not involved in the experimental procedure cell sorted and did spectral analysis from collected data.

Reporting for specific materials, systems and methods

Materials & experimental systems

n/a	Involved in the study	
☒	☐	Unique biological materials
☐	☒	Antibodies
☒	☐	Eukaryotic cell lines
☒	☐	Palaeontology
☐	☒	Animals and other organisms
☒	☐	Human research participants

Methods

n/a	Involved in the study	
☒	☐	ChIP-seq
☒	☐	Flow cytometry
☒	☐	MRI-based neuroimaging

Antibodies

Antibodies used	Polyclonal rabbit c-Fos antibody (Synaptic Systems; Cat. Number: 226003, 0.5 μg/mL. Anti mCherry Clontech (Cat. Number: 632496). lot No. 306037
Validation	Renier, N., et al. Mapping of Brain Activity by Automated Volume Analysis of Immediate Early Genes. Cell 165, 1789-1802 (2016).Renier, N., et al. iDISCO: a simple, rapid method to immunolabel large tissue samples for volume imaging. Cell 159, 896-910 (2014). Kroeger D. et al. Galanin neurons in the ventrolateral preoptic area promote sleep and heat loss in mice.

Animals and other organisms

Policy information about studies involving animals; ARRIVE guidelines recommended for reporting animal research

Laboratory animals	C-57 wildtype, Slc17a6 tm2(cre)Lowl/j and Gad2tm2(cre)zjh/J mice of 10 to 12 weeks old including male and females. Adult male Sprague Dawley rats 200-300 gr
Wild animals	N/A
Field-collected samples	Our subjects were maintained in a gated animal facility in which the inhabited cages were kept in cubicles with a reverse light cycle; the lights within the cubicle turned off at 9 AM and turned on at 9 PM. The animals were given unrestricted access to food and water and were fed according to Weill Cornell Medical College IACUC's standard diet. Additionally, the temperature in this cubicle was kept at approximately 23°C and monitored continuously.
